# Primary hydatid cyst of the thyroid glands: two case reports and a review of the literature

**DOI:** 10.1186/s13256-023-04141-3

**Published:** 2023-10-04

**Authors:** Mohammad Mostafa Safarpour, Shiva Aminnia, Amirreza Dehghanian, Roham Borazjani, Hamid Reza Abbassi, Shahram Boland Parvaz, Shahram Paydar

**Affiliations:** 1 Department of General Surgery, Shiraz University of Medical Sciences, Shiraz, Iran; 2 Student Research Committee, Shiraz University of Medical Sciences, Shiraz, Iran; 3 Molecular Pathology and Cytogenetics Division, Department of Pathology, Shiraz University of Medical Sciences, Shiraz, Iran; 4 Trauma Research Center, Shahid Rajaee (Emtiaz) Trauma Hospital, Shiraz University of Medical Sciences, Shiraz, Iran; 5 Bone and Joint Diseases Research Center, Department of Orthopedic Surgery, Chamran Hospital, Shiraz University of Medical Sciences, Shiraz, Iran

**Keywords:** Hydatid cyst, Thyroid gland, Primary, Thyroidectomy, Case report

## Abstract

**Introduction:**

Although hydatid cyst remains one of the prevalent parasitic infections in humans, hydatid cyst of the thyroid is extremely rare, even in endemic areas. Here we present two cases of thyroid hydatid cysts.

**Case presentation:**

A 35 and a 50 year-old Iranian female with a positive history of animal contact were presented with a neck lump without any compressive symptoms. A physical exam revealed neck masses that elevated with swallowing. Thyroid gland ultrasonography showed cystic thyroid lesions, and fine needle aspiration (FNA) suggested a thyroid hydatic cyst. Thyroid lobectomy and isthmectomy were done for the first patient, and near-total thyroidectomy was done for the other. The pathology report confirmed the diagnosis of a hydatid cyst. None of the patients had hydatid cysts in other sites. Patients were discharged without an antiparasitic drug, and no recurrence was detected at the six-month follow-up.

**Conclusion:**

It is necessary to consider hydatid cysts in the differential diagnosis of cystic lesions of the thyroid gland in endemic areas, especially in people with a positive history of animal contact.

## Introduction

Echinococcosis, a parasitic infection affecting humans, causes around 871,000 disability-adjusted life-years (DALYs) globally, each year [1]. Ninety-five percent of all human echinococcal diseases are caused by hydatid cyst disease (Cystic Echinococcosis) [2]. Controlling the disease costs three billion dollars annually worldwide [1].

Hydatid cyst is endemic in areas with a mild climate such as the Middle East, Southeast Asia, Mediterranean countries, and South America [3]. In Iran, the prevalence of human hydatid cyst was 4.2%, with most cases being female and living in rural regions [4].

The parasite life cycle consists of three different stages: eggs (in the environment), adult worms (in the definite host intestine), and metacestodes (in the intermediate host) [5, 6]. In the intermediate host, an oncosphere larva is released into the intestine following the ingestion of an egg. The larva then penetrates the lamina propria and gets into the liver or other organs through the blood or lymphatic system. In the internal organs, the larva forms hydatid cysts (metacestode larvae) with an inner germinal layer and an outer laminated layer containing protoscolices [7, 8].

Hydatid cyst occurs during the larval stage, and humans are the accidental intermediate hosts. The liver (65%) and the lungs (25%) are the two most frequently affected organs in humans [9]. The hydatid cyst rarely attacks the thyroid gland, even in endemic countries [2, 9–11]. The larvae can bypass the liver and lungs and settle in the thyroid gland via systemic circulation [12, 13]. The thyroid hydatid cysts usually accompany the hepatic and pulmonary cysts, and the isolated primary form is scarce, which may mimic malignancies. The growing cysts can compress the adjunct structures, eventhough most cases are asymptomatic. Moreover, anaphylactic shock and death may occur following cyst rupture [9].

All general practitioners, radiologists, surgeons, and pathologists should be aware of the presenting signs and symptoms and the paraclinical features of primary hydatid Cysts of the thyroid gland. Therefore, this study aims to present two female patients with primary hydatid cysts of the thyroid gland and highlight the clinical and paraclinical features. The Institutional Review Board and Research Ethics Committee of Shiraz University of Medical Sciences approved the study with the ethics approval number: IR.SUMS.REC.1401.185.

### Case 1

A 35-year-old Iranian female was referred for a painless anterior neck lump. The patient had noticed the swelling for about one year and stated that its size had not progressively changed. She denied a history of voice change, hoarseness, dysphagia, dyspnea, or other related symptoms. No systemic symptoms like fever, night sweats, or weight loss were reported. Past medical history was clear except for hypothyroidism which was controlled by levothyroxine since 6 years ago. She lived in an urban area and had a negative history of recent travel, occupational exposure, illicit drugs, tobacco, and alcohol use. However, she had a positive, long-lasting history of animal contact.

A non-tender, well-defined, round mass of about 2 × 3 cm in the central anterior neck was detected on physical examination. The mass was quite firm and elevated with swallowing. No warmth, erythema, or skin lesions were detected, and no cervical lymphadenopathy, exophthalmos, or other signs of hyper/hypothyroidism were seen. Head and neck, and systemic examinations were insignificant.

Laboratory data included complete blood count, thyroid function test, renal function test, blood sugar, erythrocyte sedimentation rate (ESR), and C-reactive protein (CRP) were within normal range.

Thyroid gland ultrasonography (US) showed normal-sized thyroid lobes with heterogeneous parenchymal echotexture. A cystic lesion (34 × 29 × 26 mm) with an irregular border was detected in the anterior aspect of the right thyroid lobe, containing hypoechoic solid parts.

Fine needle aspiration (FNA) under sonography guidance was done by a board-certified pathologist, and the cystic contents were completely aspirated. The specimens were reviewed using the Wright and Papanicolaou staining methods. Microscopic results showed cellular smears consisted of many hydatid cyst scolices and numerous hooks in a bloody background suggesting the diagnosis of a hydatid cyst.

**Fig. 1:**
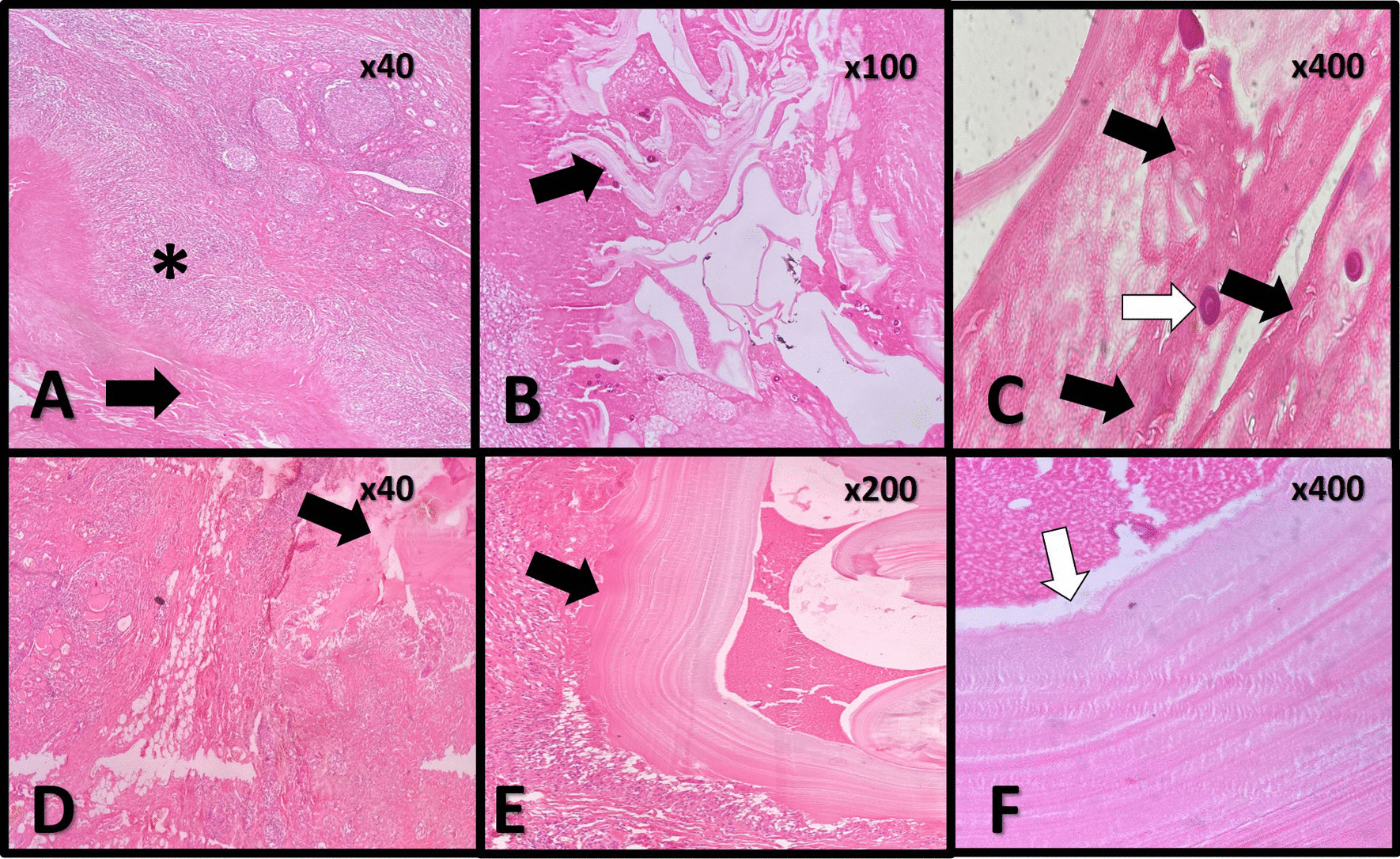
Histopathological features of the cases. **A** Histopathological section of the thyroid tissue in case 1 shows lymphocytic thyroiditis with a ruptured cystic lesion (arrow) surrounded by epithelioid histiocytes (Asterix). (× 10, H&E stain). **B** Mid-power evaluation of the cystic structure shows a ruptured laminated layer (arrow) of the hydatid cyst wall and calcified protoscolices. (× 100, H&E stain) **C** Many shiny rostellar hooklets (black arrows) in the background and a calcified protoscolex (white arrow). (X400, H&E, Closed diaphragm) **D** Histopathological section of the thyroid tissue in Case 2 shows lymphocytic thyroiditis with a ruptured cystic lesion (arrow) surrounded by epithelioid histiocytes. (× 10, H&E stain). **E** and **F** Higher power evaluation of the cystic structure shows a ruptured laminated layer (black arrow) of the hydatid cyst wall and germinal layer (White arrow). (×200, ×400 H&E stain)

Further sonographic and radiologic evaluations were negative for hydatid cysts in the other organs, including lungs and livers. Under general anesthesia, the right thyroid lobectomy and isthmectomy (by ligasure) were done. The surgical field was irrigated with hypertonic saline as a scolicidal agent [14] to avoid an anaphylactic reaction. The pathologic result confirmed the thyroid hydatid cyst (Fig. 1A-C)*.*

The postoperative course was insignificant. There was no evidence of voice change, symptomatic hypocalcemia and acute surgical site infection. The patient was discharged with levothyroxine, calcium, and calcitriol. Since no surgical contamination happened and no signs of hydatid cyst in other organs was present, antiparasitic treatment was not prescribed. Six months after the operation, no recurrence of hydatid cyst or any complication was detected, and thyroid function tests were in the normal range. Abdominopelvic ultrasonography was performed at the follow-up and was also normal.

### Case 2

A 50 year-old Iranian female presented with an anterior neck lump for about two years. The mass was painless and did not significantly grow in these years. Similar to the first case, the patient denied a history for voice changes, hoarseness, dysphagia, or other related symptoms. She had a negative history of fever, night sweats, weight loss, or other systemic symptoms. She had a past medical history of hypothyroidism and had been taking levothyroxine for 17 years. The drug and social histories were clear. As a stockbreeder, she lived in a rural area and had a long-lasting close contact with sheep, cows, goats, and dogs.

Physical examination revealed a well-defined, immobile, firm, and non-tender mass in the midline neck that elevated while swallowing. No warmth, redness, or skin lesions were present. Other physical examinations were insignificant. Moreover, the laboratory results were normal. Neck ultrasonography showed an enlarged right thyroid lobe (upper limit of normal) with inhomogeneous parenchymal echotexture and an oval solid-cystic nodule (20 × 19 × 9 mm) in the anterior aspect of the right thyroid lobe, with heterogenous intraluminal echoes. FNA was done under ultrasonography guidance, and smears of one milliliter of the bloody fluid of the cyst were prepared. Post-FNA cytology report and smear showed many scolices and hooklets of echinococcus granulomatosis. The report was negative for malignancy.

**Fig. 2:**
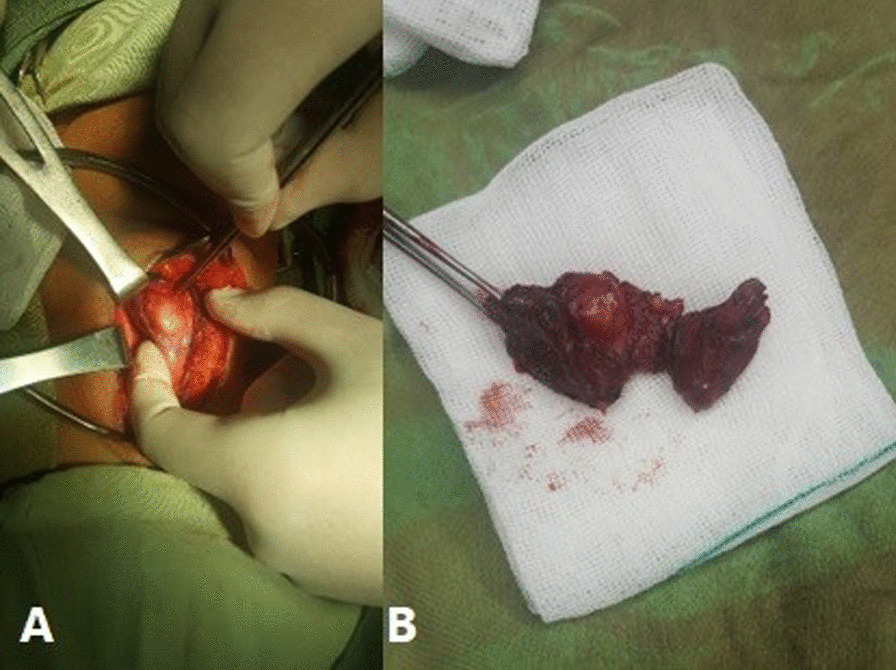
Surgical view of thyroidectomy procedure in the second case, A. whitish cyst in anterior of the neck B. thyroid gland with hydatid cyst

Near-total thyroidectomy was done (Fig. 2). The subsequent pathologic report confirmed the thyroid gland hydatid cyst (Fig. 1D–F)*.* To evaluate the presence of hydatid cysts in other internal organs, the patient underwent a chest and abdominopelvic CT scan and MRI that was insignificant.

The postoperative course was normal similar to *Case 1* since no acute symptomatic complications were detected. The patient was discharged the next postoperative day with levothyroxine and there was no need to prescribe antiparasitic agents. The follow-up visits revealed no signs of recurrence or extra-thyroid involvement or any complications.

## Discussion

The hydatid cysts frequently affect the liver and lungs. However, other organs' involvement, such as the pancreas [14], trapezius muscle [15], axillary region [16], breast [11], and ovaries [17], have been addressed. Even in highly endemic countries, including Iran, the hydatid cysts of the thyroid gland are rare [9].

The pathogenesis pathways are somewhat clear. The oncosphere larva enters the systemic circulation after bypassing or passing through the hepatic microcirculation to reach the thyroid gland (primary or secondary hydatid cysts, respectively). Although the gland is a well-nourished organ with a high blood flow, the hydatid cyst is rare due to the small diameter of its arteries [18, 19].

*Reddy and M. Thangavelu* reported the first case of thyroid involvement in 1946 [20]. Since then, several cases have been reported worldwide [2, 9, 10, 13, 21–30]. Iran is an endemic country, and some studies have reported thyroidal involvement. ***Geramizadeh*** reviewed all cases reported between 1990 to 2011; only four cases of the thyroid gland involvement have been reported. All of these cases were females and presented with compressive symptoms [11]. To the best of our knowledge, only 14 case studies (17 patients) have discussed the thyroid hydatid cyst since 2012, as detailed in Table 1. The primary hydatid cysts of the thyroid are usually asymptomatic. However, they may mimic thyroid malignancies as their size increases, and the cyst attaches to adjacent structures such as the esophagus, trachea, and recurrent laryngeal nerve, leading to compressive symptoms [31, 32]. Unlike our patients, seven studies have reported these symptoms, such as hoarseness and dyspnea, since 2012 [2, 9, 21, 23, 25, 27, 29].

**Table 1:** Ten-year review of the case reports of thyroid hydatid cysts since 2012

Author	Location	Year	no. cases	Age	Sex	Presenting signs and symptoms	Diagnosis	Management
Ghanem *et al.* [21]	Syria	2021	1	26	♀	Anterior neck mass, compressive symptoms	Histopathology	Total thyroidectomy
Salih *et al.* [22]	Iraq	2020	1	48	♀	Painless anterior neck mass,	Histopathology	Left lobectomy
Jiang *et al.* [23]	China	2019	1	54	♂	Neck swelling, intermittent dyspnea	Immunology, histopathology	Cyst removal
Eshraghi *et al.* [9]	Iran	2019	1	34	♀	Neck swelling, infrequent hoarseness	Histopathology	Left lobectomy and isthmectomy
Aydin *et al.* [24]	Turkey	2018	1	32	♀	Progressively growing neck mass	Immunology, histopathology	Total thyroidectomy
El Bousaadani *et al.* [28]	Morocco	2016	1	35	♂	Anterior neck mass	Histopathology	Thyroidectomy
Eken *et al.* [27]	Turkey	2016	1	65	♀	Growing neck mass, dyspnea	Histopathology	Total thyroidectomy
Dissanayake *et al.* [26]	USA	2016	1	44	♀	Slow-growing neck mass	FNA	Hemithyroidectomy
Bartin *et al.* [25]	Turkey	2015	1	32	♀	Growing neck mass, hoarseness	Histopathology	Total thyroidectomy
Akbulut *et al.* [2]	Turkey	2015	2	26	♀	neck swelling and pain	Histopathology	Total thyroidectomy
				57	♀	sore throat and neck swelling	Histopathology	Total thyroidectomy
Dey *et al.* [10]	India	2014	1	30	♀	Neck lump	FNA	Albendazole for 28 days(conservative)
Yilmaz *et al.* [13]	Turkey	2013	3	18	♂	Neck swelling	Indirect hemagglutinin, histopathology	Albendazole and left lobectomy and isthmectomy
				25	♀	neck pain	Histopathology	Total thyroidectomy
				21	♂	neck swelling	Histopathology	Total thyroidectomy
Sersar *et al.* [30]	Saudi Arabia	2013	1	48	♀	Neck cyst	Histopathology	Albendazole for six weeks, then thoracotomy and thyroidectomy
Oksuz *et al.* [29]	Turkey	2013	1	23	♂	Hoarseness	Histopathology	Subtotal thyroidectomy

NO. Cases,  number of involved cases; ♀,  female; ♂,  male; FNA, fine needle aspiration

Our patients did not report any significant changes in the size of the masses. Generally, the hydatid cysts are not known as rapidly growing neck masses, although in some cases, they may progressively grow [24–27]. Moreover, the primary hydatid cysts are usually solitary cystic lesions affecting a single thyroid lobe; however, more than one cyst can also be detected. In the case report by Aydin *et al.*, two cysts in the two thyroid lobes were found [24]. Similar to most previous studies, a solitary cyst in the right thyroid lobe was detected in our patients.

The diagnosis is based on clinical and paraclinical analyses such as ultrasound and FNA findings. However, most cases are diagnosed after surgery, and post-surgical pathology remains the gold standard of diagnosis [33, 34]. The literature showed that almost all cases were diagnosed through post-surgical histopathology findings. However, the patient management in two studies reported in the USA [26] and India [10] was solely based on FNA since 2012 (Table 1). In both cases, the scolices/protoscolices hooklets were seen in FNA. We detected the same findings in the FNA of our two patients; however, we decided to examine the histopathological features of the removed gland for further confirmation and to rule out any concomitant malignancies.

When a hydatid cyst is expected, using FNA is controversial regarding the likelihood of cyst rupture. Various complications from mild allergic to anaphylaxis reactions have been reported following the FNA of the hydatic cysts [35–37]. The most common complications are anaphylaxis following cyst rupture and abscess formation [2, 12]. Neither of our cases reported in this study showed complications related to cyst rupture. Of course, in both cases, after FNA, the liquid contents inside the cyst were completely drained. Moreover, the surgical field was irrigated with hypertonic saline during the operation as the previous studies have recommended using hypertonic saline to prevent the occurrence of intra-operative anaphylaxis [38].

In our study, patients presented with neck lumps without any other symptoms. The US was the first modality for evaluating the neck mass, which showed cystic lesions with intraluminal solid parts in both cases. The US helps distinguish solid or cystic lesions of the neck, and if a detachment of the membrane of the cyst with a multilocular appearance exists, it can suggest a hydatid cyst [39]. Unfortunately, in our cases, the ultrasound findings did not indicate the hydatid cysts. Since the US is an operator-dependent diagnostic tool, high clinical suspicion may help the radiologist detect hydatid cysts.

Surgical resection is the treatment of choice, and antiparasitic medications like Mebendazole or Albendazole may be administered when there is a contraindication for surgery [40, 41]. According to the current literature, only three cases have been managed using antiparasitic medications. A 30-year-old female reported by Dey *et al.* received only Albendazole treatment (400 mg/day) for 28 days without any subsequent surgery [10]. One of the cases reported by Yilmaz *et al.* received mixed treatment. She was an 18-year-old female who received Albendazole, followed by left lobectomy and isthmectomy [13]. The last patient was a 48-year-old female with bilateral pulmonary and thyroid cysts. She received Albendazole for six weeks but did not improve. The thoracotomy and bilateral lobectomy removed the complicated cysts [30]. We did not use antiparasitic drugs, and surgical removal was done for both cases. Of note, they have no signs of recurrence or other remote organ involvement at the 6-month follow-up.

## Conclusion

Considering the endemicity of hydatid cysts in Iran [4] and the Middle East [13, 24, 29, 35] and the occurrence of thyroid involvement, physicians and stakeholders must know about the common epidemiological, clinical, and paraclinical features. Moreover, it is essential to consider hydatid cysts in endemic areas as one of the differential diagnoses for cystic lesions in the thyroid. In conclusion, we reported two females with primary thyroid hydatid cysts. They had no local or systemic symptoms. They were treated through thyroidectomy, and no recurrence was detected at the 6-month follow-up. The current study emphasizes that high clinical suspicion is needed to differentiate thyroid hydatid cysts from other cystic lesions in regions.

## Data Availability

Not applicable.

## Abbreviations

DALYs: Disability-Adjusted Life-Years
ESR: Erythrocyte sedimentation rate
CR: C-reactive protein
US: Ultrasonography
FNA: Fine needle aspiration
H&E: Haematoxylin and Eosin
